# *Borrelia burgdorferi*-Induced Changes in the Class II Self-Immunopeptidome Displayed on HLA-DR Molecules Expressed by Dendritic Cells

**DOI:** 10.3389/fmed.2020.00568

**Published:** 2020-09-16

**Authors:** Maria G. Gutierrez-Hoffmann, Robert N. O'Meally, Robert N. Cole, Eleni Tiniakou, Erika Darrah, Mark J. Soloski

**Affiliations:** ^1^Lyme Disease Research Center, Johns Hopkins University School of Medicine, Baltimore, MD, United States; ^2^Division of Rheumatology, Johns Hopkins University School of Medicine, Baltimore, MD, United States; ^3^Mass Spectrometry and Proteomics Facility, Department of Biological Chemistry, Johns Hopkins University School of Medicine, Baltimore, MD, United States

**Keywords:** Borrelia, immunopeptidome, HLA-DR, dendritic cells, MHC class II

## Abstract

The MHC class II antigen processing and presentation pathway has evolved to derive short amino acid peptides from proteins that enter the endocytic pathway, load them onto MHC class II molecules and display them on the surface of antigen presenting cells for recognition by CD4^+^ T cells. Under normal circumstances, peptides bound to MHC class II molecules are derived from host (self) proteins and not recognized by T cells due to tolerance mechanisms. Pathogens induce significant changes in the biology of antigen presenting cells, including upregulation of MHC processing and presentation. We therefore hypothesized that exposure to pathogens may alter the repertoire of self-peptides bound to MHC class II molecules. To test this hypothesis, we isolated monocyte-derived dendritic cells from healthy subjects, exposed them to the TLR-2 agonist lipoteichoic acid or live *Borrelia burgdorferi*, the causative agent of Lyme disease, and isolated and characterized HLA-DR associated peptides using mass spectrometry. Our results show that lipoteichoic acid-stimulated, *B. burgdorferi*-stimulated and unstimulated monocyte-derived dendritic cells largely derive their self-peptides from similar overlapping sets of host proteins. However, lipoteichoic acid and *B. burgdorferi* stimulation promote the processing and presentation of new sets of HLA-DR associated self-peptides derived from unique protein sources. Examination of processes and compartments these proteins reside in, indicate that activation of monocyte-derived dendritic cells changes the range of host self-proteins available for processing and presentation on MHC class II molecules. These findings reveal that the HLA-DR-bound self-immunopeptidome presented by mo-DCs is dynamic in nature and changes with activation state reflective of cellular function. In addition, among the repertoire of self-peptides bound to HLA-DR are several epitopes known to be recognized by autoreactive T cells. These studies are relevant to our basic understanding of pathogen-induced changes in monocyte-derived dendritic cell function, and the mechanisms involved in infection-induced autoimmune illnesses such as Lyme arthritis.

## Introduction

Lyme disease is an inflammatory illness initiated by infection with the *Borrelia burgdorferi* spirochete following a bite from an infected *Ixodes* tick (1). Over the last four decades, the number of Lyme disease cases has risen sharply, and it is now the most common vector-borne disease in the United States with over 300,000 cases each year (2). Symptoms of early Lyme disease can range from erythema migrans alone to systemic toxicity with signs of disseminated infection. A number of patients with undetected and untreated early Lyme disease will develop late-onset musculoskeletal (Lyme arthritis) or neurological symptoms (Neuroborreliosis) (3). While the acute infection and late-onset disease can be controlled by antibiotic therapy, in a subset of patients, arthritis with inflammation can be antibiotic-refractory (4). This outcome has been termed post-infectious Lyme arthritis, with autoimmune processes presumed to play a major role and although controversial, bacterial persistence cannot be excluded from contributing to the development of the illness (5). Further, 10–20% of patients treated for early Lyme disease develop Post-Treatment Lyme Disease Syndrome (PTLDS), a condition with unknown pathophysiology that may have an autoimmune component (6–8). Clearly, infection with *B. burgdorferi* triggers poorly understood immune processes, and considering the rising incidence of Lyme disease as well as the complexity of disease outcomes, a deeper understanding of the immune-mediated process triggered by Borrelia is needed.

Dendritic cells (DCs) are major drivers of the adaptive immune response against pathogens (9). These cells are present at strategic sites of pathogen entry such as the skin (10). At homeostasis, immature DCs are tissue resident, highly phagocytic, and have a constitutive antigen processing and presentation pathway expressing low levels of major histocompatibility (MHC) molecules, loaded with self-peptides. Upon encounter with a pathogen, a range of surface and intracellular pattern recognition receptors signal a complex maturation program, which leads in part, to the down regulation of phagocytic activity, the upregulation of relevant co-stimulatory molecules, as well as an increase in antigen processing, presentation, and MHC molecule expression (11). This results in a mature dendritic cell that expresses MHC molecules loaded with pathogen-derived peptides (11). These mature cells then relocate to the T cell rich areas of draining lymph nodes initiating a T cell dependent host response (12).

The infection route *via* tick bite introduces the Borrelia bacterium into the skin, a site where dendritic cells reside (10). Given the importance of DCs in the initiation of T cell-dependent responses and the key role for CD4^+^effector T cells in human Lyme disease pathogenesis, we investigated the consequences of *B. burgdorferi* interaction with human monocyte-derived dendritic cells (mo-DCs) *in vitro*. The results show that *B. burgdorferi* uniquely alters the self-peptide repertoire. These peptides are derived from a new set of proteins which occupy novel compartments and/or pathways. These observations will be discussed in the context of human Lyme pathogenesis and infection-induced immunity.

## Materials and Methods

### *Borrelia burgdorferi* Culture

*Borrelia burgdorferi* strains A3 (kindly provided by Dr. Utpal Pal, University of Maryland), B31 (ATCC 35210), and B31-5A19 (kindly provided by Dr. Monica Embers, Tulane University) were grown from frozen stocks in 5 mL tightly closed conical tubes of BSK-II incomplete culture medium made in house [9.82 g CMRL-1066 without L-glutamine, 5.0 g neopeptone, 2.0 g yeastolate, 6.0 g HEPES, 5.0 g glucose, 0.7 g sodium citrate dihydrate, 0.8 g sodium pyruvate, 0.4 g N-acetyl-D-glucosamine, 2.2 g sodium bicarbonate, 50.0 g bovine serum faction V (albumin, bovine serum), 1 L of deionized water], supplemented with 6% rabbit serum and 7% gelatin for 14 days. Bacterial cultures were grown at 34°C, 5% CO_2_, and >95% humidity and used between 14 and 21 days old while in the logarithmic growth phase. For inoculum preparation, 1–5 mL of each *B. burgdorferi* strain was pelleted at 6000 RCF for 8 min at room temperature. Aliquots were resuspended in 1 mL of phosphate buffered saline (PBS), bacteria were counted using dark-field microscopy, pelleted and resuspended to the desired concentration in RPMI 1640 medium with 10% fetal bovine serum.

### Monocyte-Derived Dendritic Cell Culture

Deidentified leukopacks classified as medical excess from consented, healthy, anonymous plasma/platelet donors were sourced from the Ann Arundel Medical Center in Annapolis, MD. Immunogenetic genotype information from the donor cohort was performed by the Johns Hopkins Immunogenetics Laboratory and is available in Table 1.

**Table 1:** Healthy donor identifiers, experimental details, and statistical results generated with PEAKS X from individual LC-MS/MS experiments.

**Healthy Donor**	**Stimulus**	**Peptide-Spectrum Matches**	**Peptide Sequences**	**Proteins**	**Peptide −10lgP**	**False Discovery Rate (%)**	**HLA-DRB1 Locus**	
							**Allele 1**	**Allele 2**
190125	None	755	464	451	≥20	2.60	N/A	N/A
	Lipoteichoic acid	N/A	N/A	N/A	N/A	N/A		
	*Borrelia burgdorferi*	2001	987	1081	≥20	0.90		
190506	None	572	411	442	≥20	0	DRB1*03:01/ 03:147	DRB1*03:02
	Lipoteichoic acid	601	440	546	≥20	0.2		
	*Borrelia burgdorferi*	1045	725	657	≥20	0.4		
190529	None	1439	731	671	≥20	0.3	DRB1*11:01	DRB1*04:01
	Lipoteichoic acid	1920	1205	1045	≥20	0.5		
	*Borrelia burgdorferi*	1532	949	830	≥20	0.5		
190726	None	N/A	N/A	N/A	N/A	N/A	DRB1*03:01/ 03:147	DRB1*07:01
	Lipoteichoic acid	1238	777	973	≥20	0.2		
	*Borrelia burgdorferi*	1723	1033	1103	≥20	0.2		
191016	None	4343	3111	2285	≥20	0	DRB1*01:01	DRB1*04:01
	Lipoteichoic acid	4216	2928	2141	≥20	0.1		
	*Borrelia burgdorferi*	4138	2999	2153	≥20	0.1		
191114	None	949	647	754	≥20	0.8	DRB1*01:01	DRB1*16:01
	Lipoteichoic acid	1614	1116	980	≥20	0.1		
	*Borrelia burgdorferi*	2302	1595	1262	≥20	0.1		
191202	None	221	156	195	≥20	0.9	DRB1*11:01	DRB1*15:01
	Lipoteichoic acid	552	393	468	≥20	0		
	*Borrelia burgdorferi*	659	476	490	≥20	0.5		
200212	None	1651	1109	1532	≥20	1	DRB1*11:03	DRB1*13:01
	Lipoteichoic acid	418	179	241	≥20	2.6		
	*Borrelia burgdorferi*	1025	520	530	≥20	0.3		
200218	None	3645	2341	1969	≥20	0.2	DRB1*01:01	DRB1*15:01
	Lipoteichoic acid	4302	2696	2064	≥20	0.3		
	*Borrelia burgdorferi*	4009	2647	1900	≥20	0.3		

Peripheral blood mononuclear cells (PBMCs) were isolated from donor leukopacks by Ficoll-Paque density gradient centrifugation from ~100 mL of leukocytes isolated by leukapheresis. CD14 MicroBeads (MACS Miltenyi Biotec) were used for CD14^+^ selection according to manufacturer's instructions and isolated monocytes were differentiated into mo-DCs in Mo-DC Differentiation Medium (MACS Miltenyi Biotec) at 10^6^ cells/ml for 7 days as described previously (13). Cells were incubated at 37°C, 5% CO_2_, and >95% humidity. At the end of the culture period the resulting cell population was routinely >95% CD14^−^/CD11c^+^, as assessed by flow cytometry.

### *In vitro* Activation of Monocyte-Derived Dendritic Cells for Flow Cytometry Analysis

We performed a natural antigen processing assay (NAPA) as described previously (13). Briefly, immature mo-DCs were seeded in 6-well plates at a density of 1 × 10^6^ cells in 1 mL of RPMI 1640 and 10% fetal bovine serum, supplemented with 2 mM L-glutamine and 20 μM ß-mercaptoethanol in duplicate. Immature mo-DCs were left at rest or stimulated with 1 μg/mL purified lipoteichoic acid (LTA) from *Staphylococcus aureus* (InvivoGen, San Diego, CA) or live *B. burgdorferi* strains A3 or B31 at multiplicities of infection of 1 or 10 bacteria per cell for 8 and 24 h. Cells were incubated at 37°C, 5% CO_2_, and >95% humidity. At the end of the incubation period, all samples were harvested into a microcentrifuge tube, pelleted at 1,200 rpm at room temperature for 3 min. Cells were washed with PBS/EDTA (PE) Buffer and pelleted at 1,200 rpm at room temperature for 3 min. Cells were stained with CD14-PE (BD Pharmingen), CD11c-PerCP (BioLegend), HLA-DR-BV510 (BD Horizon), and Blue Fluorescent Reactive Dye (Life Technologies) for 15 min in BV Buffer (BD Horizon). At the end of the incubation period, cells were washed twice in PBS and resuspended in 100 μL PBS for flow cytometry analysis in a BD FACSAria II flow cytometer (BD Biosciences). All data was gated on CD14^−^/ CD11c^+^ monocyte derived dendritic cells.

### *In vitro* Activation of Monocyte-Derived Dendritic Cells for Immunopeptidome Isolation

Immature mo-DCs were seeded in T-25 cell culture flasks at a density of 2–15 × 10^6^ cells in 7.5 mL of RPMI 1640 and 10% fetal bovine serum, supplemented with 2 mM L-glutamine and 20 μM ß-mercaptoethanol. Immature mo-DCs were stimulated with 1 μg/mL purified LTA or live *B. burgdorferi* strains A3 or B31-5A19 at a multiplicity of infection of 10 bacteria per cell for 24 h. Cells were incubated at 37°C, 5% CO_2_, and >95% humidity. At the end of the incubation period, naturally processed and presented peptides were isolated from HLA-DR molecules by immunoprecipitation using a natural antigen processing assay (13). Briefly, cells were harvested and pelleted at 1,200 rpm for 10 min at 4°C. Culture flasks were washed with cold PE buffer, adherent cells were lifted, and this solution was used to pellet cells. Cells were resuspended in 400 μL/2 × 10^6^ cells in lysis buffer (1% CHAPS, pepstatin, leupeptin, chymostatin, antipain, PMSF, and EDTA) and lysed for 1 h at 4°C in a rocking table. Cellular debris was removed by centrifugation at 10,000 RCF for 15 min to clear supernatant twice. Twenty μg of the anti-HLA-DR antibody L243 (purified from ATCC HB-55 hybridoma) was added to each sample and incubated overnight at 4°C. One hundred μL of Rec-Protein G-Sepharose 4B conjugate (Thermo Fisher Scientific) slurry was added to the antigen-antibody complex and incubated with gentle mixing for 2 h at room temperature. The agarose-antibody-antigen complex was washed with 500 μL of a 20 mM Tris, pH 7.4 and 150 mM NaCl solution. Peptides were eluted from the bead-antibody complex in 100 μL of 1% trifluoroacetic acid (TFA) and isolated using C18 spin columns (Thermo Fisher Scientific) according to manufacturer's instructions.

### Liquid Chromatography-Mass Spectrometry/Mass Spectrometry (LC-MS/MS)

Samples were processed in Waters Oasis MAX (Mixed-mode Anion eXchange) 96-well microelution plates using a Waters Positive Pressure-96 Processor prior to mass spectrometry analysis. Each Oasis well was conditioned with 100 μL of 4% H_3_PO_4_, samples were diluted 1:1 with 4% H_3_PO_4_, and transferred to the microelution plate. Pressure was applied to concentrate the peptides on the MAX phase, washed twice with 50 μl of 5% NH_4_OH followed by two 50 μL washes of 20% acetonitrile. The flow through was discarded and a new clean 96-well plate was placed under the Oasis MAX plate. Peptides were eluted from the MAX phase with two 50 μL aliquots of 75% acetonitrile containing 1% TFA. Eluted peptides were dried down by speed vacuum centrifugation and reconstituted in 2% acetonitrile, 0.1% formic acid. Peptides were injected into a trap column, eluted over a 90 min, 2–90% acetonitrile gradient containing 0.1% formic acid at 300 nL per minute. Columns were packed in house using a New Objective 75 μm ID PicoFrit with ReproSil-Pur C18 AQ 3 μm stationary phase. Peptides were eluted with a Thermo Fisher Easy-nanoLC system interfaced with a Thermo Scientific Orbitrap Fusion Lumos Tribrid Mass Spectrometer. Peptides were analyzed with a data dependent 3 s cycle fragmentation method for the highest abundant precursors. Survey and MS2 scans were performed at 120,000 and 30,000 resolution, respectively. The mass spectrometry.raw files were searched with the PEAKS X software against the *Homo sapiens* RefSeq database protein sequences with no enzyme designation and allowing for variable modification of methionine oxidation and asparagine or glutamine deamidation. The resulting peptide identifications were filtered at a −10lgP-value of 20 using the PEAKS decoy-fusion algorithm.

### Data Analysis

For flow cytometry analysis, statistical significance was calculated by comparing the 0 h unstimulated median MFI (Median Fluorescence Intensity) vs. median MFIs from each condition using the One-way ANOVA (*p* < 0.0001) statistical test followed by Dunnett's multiple comparisons test (^****^p < 0.0001) using GraphPad Prism version 8.4.2.

The BioVenn web application (https://www.biovenn.nl) was used to generate area-proportional Venn diagrams to visualize overlap of all identified parent proteins using the SVG Only display option with the print numbers option selected and both the absolute nrs and percentages options selected as well (14). Corresponding ID lists of proteins found in common between all stimuli (Unstimulated, LTA, and *B. burgdorferi*) and those uniquely found in only one stimulus (Unstimulated or LTA or *B. burgdorferi*) were exported from the BioVenn web application using the Current Image Statistics lists.

The Advanced Biomedical Computing Center's biological DataBase network (bioDBnet, https://biodbnet-abcc.ncifcrf.gov/db/db2db.php) db2db Database to Database Conversions version 2.1 was used to convert non-redundant RefSeq Protein Accession numbers (input) exported from BioVenn's Current Image Statistics lists into UniProt Accession identifiers (output) (15).

The Laboratory of Human Retrovirology and Immunoinformatics's Database for Annotation, Visualization and Integrated Discovery (DAVID) version 6.8 Functional Annotation Clustering tool was used to cluster genes into Gene Ontology (GO) terms (16, 17). Individual ID lists of Uniprot Accession numbers were individually uploaded to DAVID (Step 1) under the Uniprot_Accession Identifier (Step 2) as a Gene List (Step 3). DAVID's default settings were unchecked in order to individually select GO terms BP_ALL (BP, Biological Processes) and CC_ALL (CC, Cellular Compartments) and the Functional Annotation Clustering tool was selected. Binning of all gene sets was rerun at a high classification stringency and an EASE score of 0.1 as the Enrichment Threshold. All other options were left at default settings. DAVID's algorithm assigns Enrichment Scores to each Annotation Cluster by calculating the geometric mean in -log scale of the cluster members' *p*-values. The *p*-values assigned to each GO term in the Annotation Clusters equals the Fisher Exact/EASE Score assigned to each GO term. Kappa statistics and fuzzy heuristic clustering measure the degree of common genes between two terms and groups similar terms according to kappa values (16, 17). Listings of enriched clusters were downloaded from DAVID's web application and bar graphs displaying the top 20 enrichment clusters (if DAVID thresholds were met) and corresponding *p*-values were graphed using GraphPad Prism version 8.4.2.

Core sequences of identified integrin α-M precursor peptides were predicted with the Denmark Technical University (DTU) NetMHCIIpan-3.2 server using default settings (18). Predicted core sequences were then submitted to DTU's Seq2Logo web-based sequence logo generation method (19). Kullback-Leibler type logos for specific *HLA-DRB1* alleles were generated using the Hobohm1 clustering method with a 0.63 clustering threshold, 200 pseudo counts, and 50 stacks per line. Amino acid coloring scheme was set to Seq2Logo defaults.

## Results

### *Borrelia burgdorferi* Induces the Upregulation of Cell Surface HLA-DR

**Figure 1:**
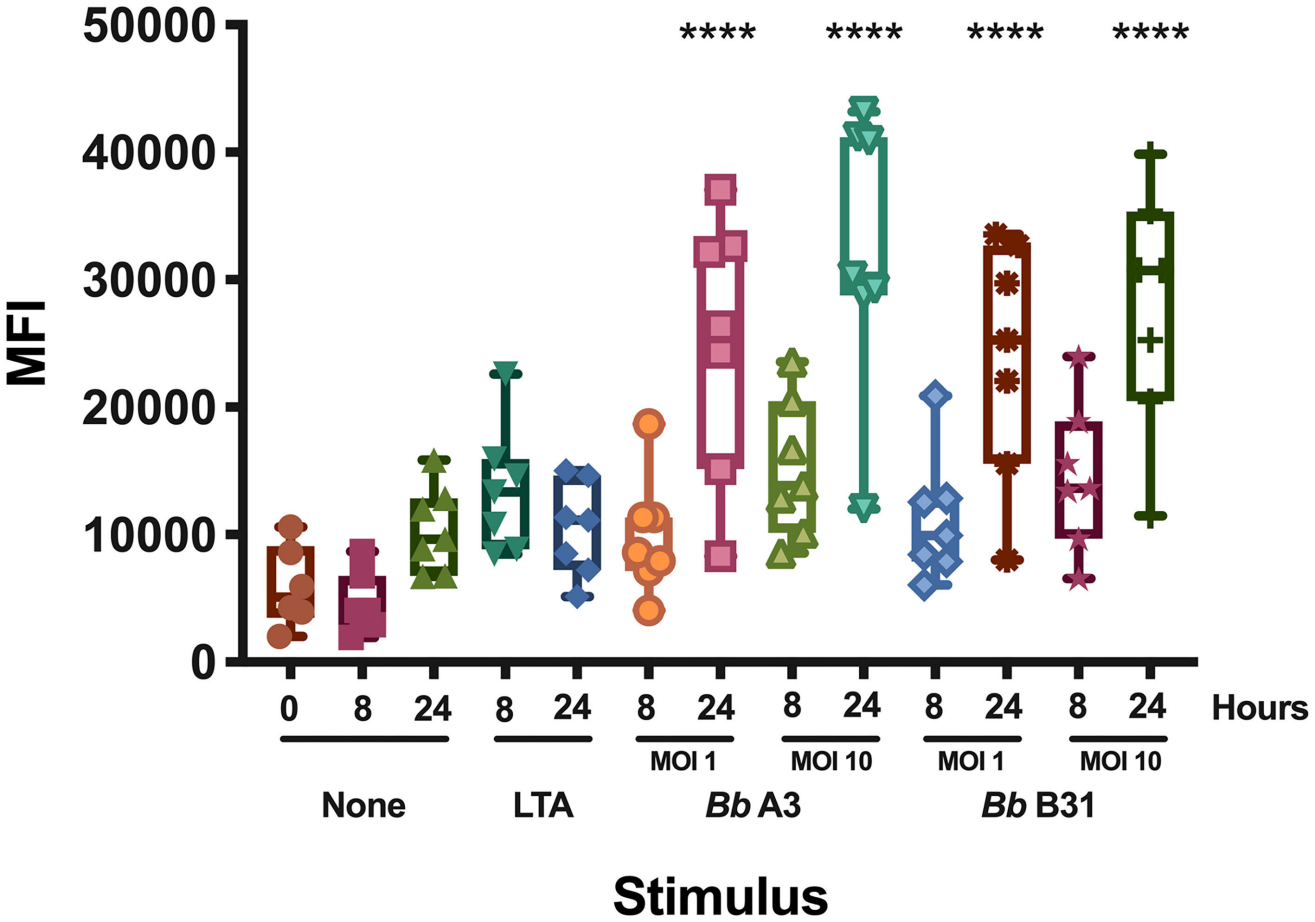
Cell surface expression of HLA-DR in monocyte-derived dendritic cells. Expression of HLA-DR on mo-DCs left unstimulated or stimulated with LTA or live *B. burgdorferi* was measured at baseline, 8- and 24-h post incubation by flow cytometry. Statistical significance was calculated by comparing the 0 h unstimulated median MFI (Median Fluorescence Intensity) vs. median MFIs from each condition using the One-way ANOVA (*p* < 0.0001) statistical test followed by Dunnett's multiple comparisons test (****p < 0.0001) using GraphPad Prism version 8.4.2.

Dendritic cells play a key role in the initiation of the human adaptive immune response against invading pathogens. Engagement of the T cell receptor by MHC class II HLA-DR molecules loaded with a foreign antigen peptide on DCs is an essential signal necessary to initiate T cell activation (12). Accordingly, we investigated changes in cell surface expression of HLA-DR in mo-DCs at rest or upon stimulation with the Toll-like receptor-2 agonist LTA from *Staphylococcus aureus* or with live *B. burgdorferi* at baseline, 8, and 24 h post treatment. Our results showed that *B. burgdorferi* strains A3 and B31 at multiplicities of infection of 1 or 10, induce upregulation of HLA-DR on the surface of mo-DCs in a time and dose dependent manner when compared to unstimulated mo-DCs and those stimulated with LTA (Figure 1). These results indicate that *B. burgdorferi* can supply the required signals to initiate dendritic cell maturation.

### Parent Protein and Supporting Peptide Characteristics

**Figure 2:**
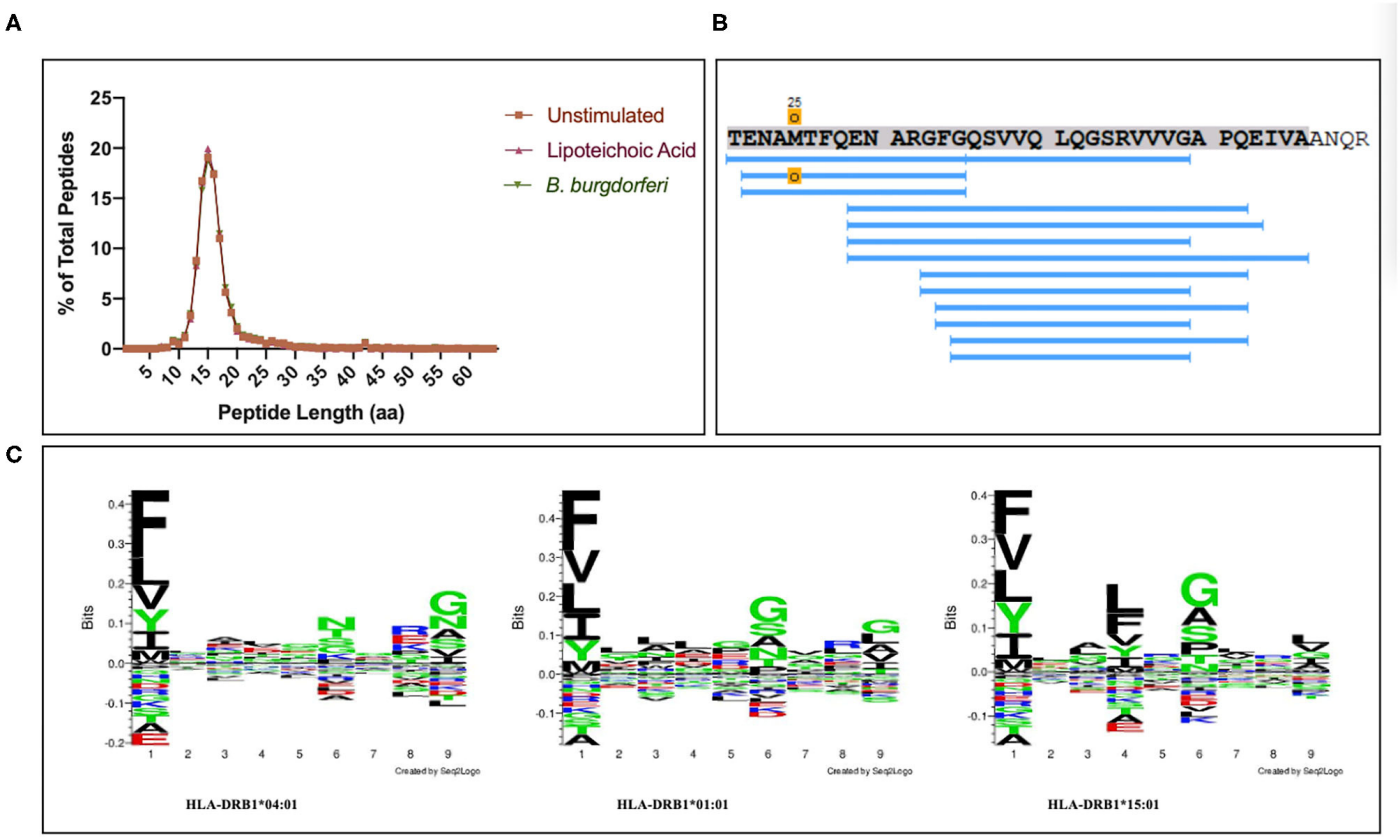
Identified peptides meet characteristics of MHC class II processed and HLA-DR presented peptides. **(A)** Percent of all peptides identified by LC-MS/MS from mo-DCs left at rest or stimulated with LTA or live *B. burgdorferi* for 24 h, based on peptide length. GraphPad Prism version 8.4.2 was used for graphical representation. **(B)** Representative nested set of peptides derived from the protein integrin α-M precursor (residues 21–56, donor 191016) characteristic of MHC class II processed proteins. **(C)** Seq2Logos of peptides derived from integrin α-M precursor from donors 190529, 191016, and 200218 with *HLA-DRB1* alleles 04:01, 01:01, or 15:01. Seq2Logo default amino acid colors D and E red; N, Q, S, G, T, and Y green; R, K, and H blue; and unassigned amino acids black.

We set out to define the mo-DC immunopeptidome after exposure to *Borrelia burgdorferi*. Monocyte-derived dendritic cells differentiated from PBMCs from 9 healthy donors (Table 1), were used as a source of APCs. The mo-DCs were either left unstimulated, stimulated with LTA or exposed to live *B. burgdorferi* for 24 h. The peptide-HLA-DR complexes were isolated by immunoprecipitation and peptides eluted from the HLA-DR groove were processed for LC-MS/MS identification. We set a stringent threshold for peptide identification in the PEAKS X software to −10lgP-values ≥ 20 (~*p* ≤ 0.01) in order to filter the identified human peptides at a 1% false discovery rate using the PEAKS decoy-fusion algorithm. Using this approach, we identified HLA-DR-associated peptides from every donor. The number of peptide-spectrum matches ranged from 221 to 4,343, with 156 to 2,999 peptide sequences detected (Table 1). We next identified the human parent proteins for all detected peptides. Across all subjects and conditions, we found 4,146 parent proteins as peptide sources in unstimulated mo-DCs, 4,070 parent proteins in mo-DCs stimulated with LTA, and 4,038 parent proteins in mo-DCs stimulated by *B. burgdorferi*. Peptides identified from self-proteins matched common characteristics of MHC class II presented peptides, with the average length of the most abundant peptides ranging between 9 and 26 amino acids (Figure 2A) and nested peptide sets at specific regions within the parent protein (Figure 2B). Using peptides derived from integrin α-M precursor, a peptide donor in all subjects, we generated amino acid binding motifs and sequence profiles using the Seq2Logo sequence logo generator. The constructed logo matched published MHC II binding motifs based on the individual's *HLA-DRB1* alleles (Figure 2C) (20). In general, peptides that bind class II MHC molecules share a hydrophobic residue at positions 1 and 9 [phenylalanine (F), tyrosine (Y), leucine (L), valine (V), isoleucine (I), alanine (A), glycine (G)], a negatively charged residue at position 4 [aspartic acid (D) or glutamic acid (E)], and an inclination for a basic residue at position 6 [lysine (K), arginine (N), histidine (H), glutamine (Q) or asparagine (N)] (21). Overall, these results validate the observed characteristics of the peptides identified in our assays as *bona fide* class II MHC-processed and presented epitopes.

### Identification of Source Proteins for HLA-DR-Bound Peptides: Common and Unique Features Under Different Conditions

**Figure 3:**
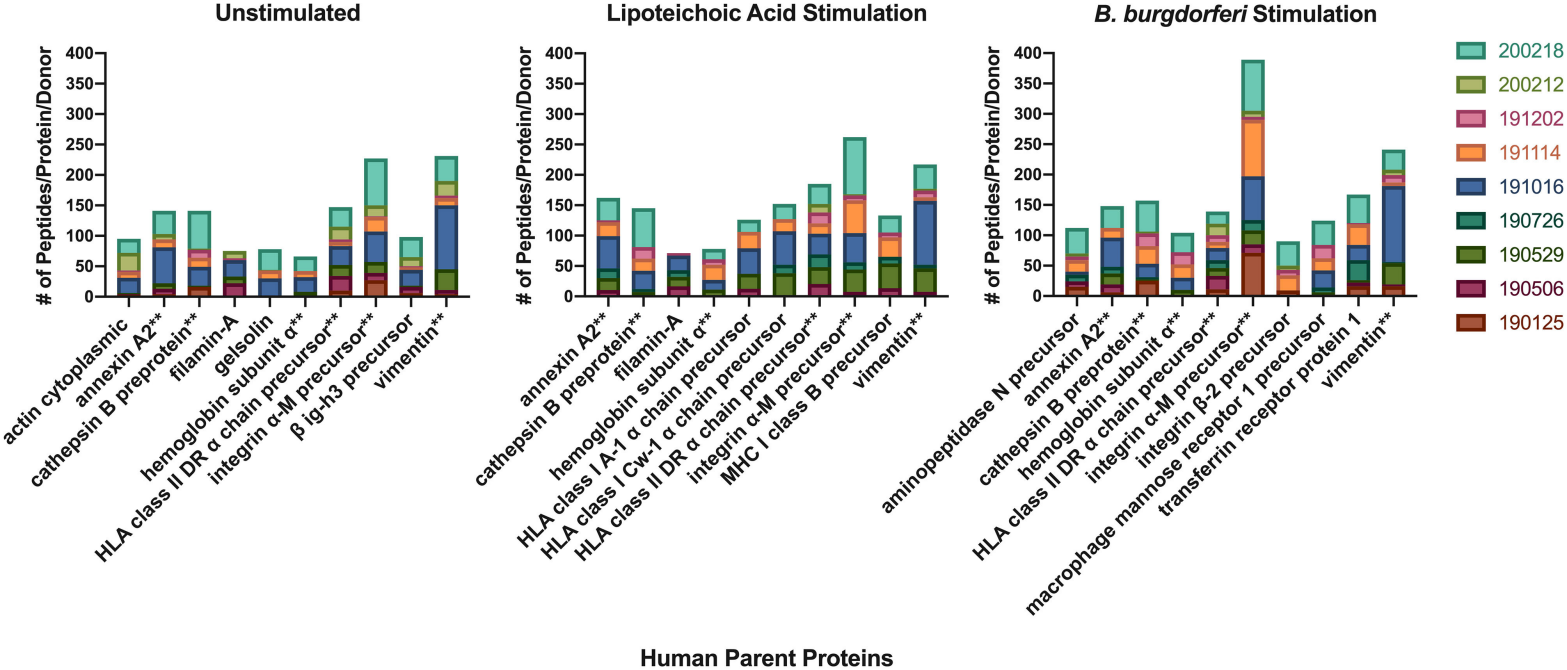
Most abundantly presented parent proteins in all stimuli. Number of identified peptides from the top 10 parent proteins from healthy mo-DCs left at rest (left panel) or stimulated with LTA (middle panel) or live *B. burgdorferi* (right panel) for 24 h. GraphPad Prism version 8.4.2 was used for graphical representation. **Denotes parent proteins identified in all stimuli.

**Figure 4:**
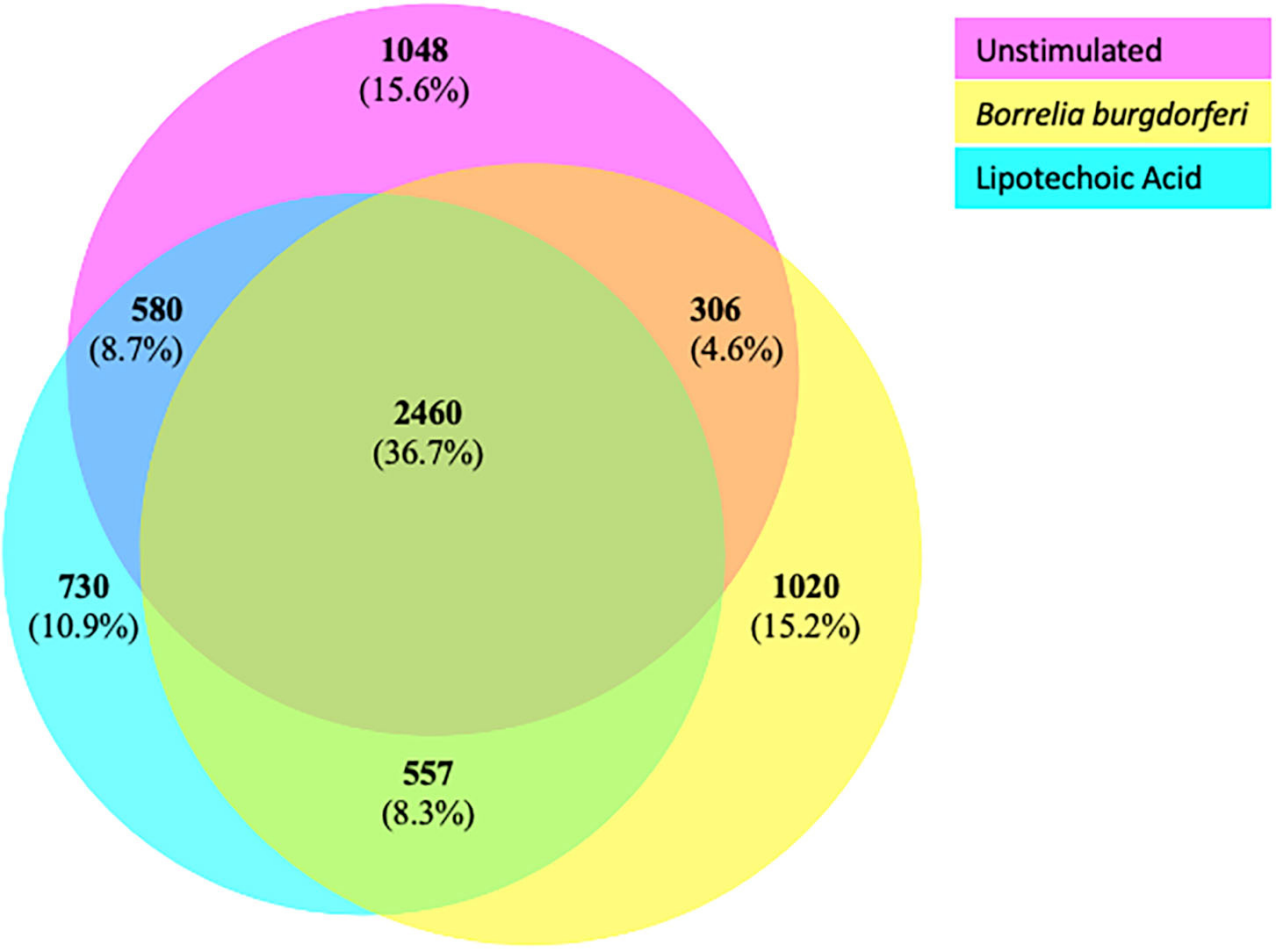
Common and exclusive parent proteins presented in all stimuli. BioVenn diagram illustrating overlap and exclusivity in source proteins identified by LC-MS/MS from mo-DCs left at rest (pink circle) or stimulated with LTA (aqua circle) or live *B. burgdorferi* (yellow circle) for 24 h.

We next characterized the source proteins of identified HLA-DR bound peptides in all subjects and all conditions. The most commonly presented proteins in all stimuli were integrin α-M, vimentin, annexin A2, cathepsin B isoform 1 preprotein, HLA class II DR α chain precursor, hemoglobin subunit α, filamin-A, aminopeptidase N precursor, actin cytoplasmic, HLA class I A-1 α chain, HLA class I Cw-I α chain precursor, major histocompatibility complex class I B precursor, transferrin receptor protein, macrophage mannose receptor 1 precursor, transforming growth factor beta-induced protein ig-H3, and gelsolin (Figure 3 and Table 2). Peptides from all parent proteins that were commonly identified were found in at least 4 out of the 9 (44%) healthy subjects. Globally, we observed significant overlap of source proteins, with 2,460 proteins (36.7%) contributing peptides for presentation in all donors and all stimuli, while smaller numbers of proteins were shared between two stimulation conditions (Figure 4). Interestingly, 1,048 (15.6%), 730 (10.9%), and 1,020 (15.2%) of all identified source proteins were exclusively presented by immature mo-DCs, LTA-stimulated mo-DCs and *B. burgdorferi*-stimulated mo-DCs, respectively (Figure 4). Individual donors paralleled this trend regardless of HLA-DR genotype (Supplementary Figure 1). This latter observation indicates that monocyte-derived dendritic cells will display self-peptides derived from unique protein sources depending on their physiological state.

**Table 2:** Most abundantly identified parent proteins presented by HLA-DR molecules detected in all donors and conditions.

**Parent protein**	**Accession number**	**Stimulus**	**# of Donor Peptides**								
			**190125**	**190506**	**190529**	**190726**	**191016**	**191114**	**191202**	**200212**	**200218**
Actin cytoplasmic 1 & 2	NP_001092.1 NP_001186883.1	None	3	1	2	–	25	10	2	29*	23
		LTA	–	1	6	3*	28*	12	5	–	28*
		*Bb*	4	–	7	–	34	20*	–	–	26
Aminopeptidase N precursor	NP_001141.2	None	3	4	–	–	9	3	5	8	39
		LTA	–	3	–	7	12	16	6	–	51
		*Bb*	15	9	–	11	5	19	6	5	42
Annexin A2 (isoform 1 & 2)	NP_001002858.1 NP_001002857.1	None	4	9	9	–	59	13	–	9	38
		LTA	–	10	20	16	53	23	3	–	37
		*Bb*	6	13	18	11	48	16	–	–	36
Cathepsin B isoform 1 preprotein	NP_001899.1	None	16	–	2	–	31	15	13	2	62
		LTA	–	–	7	5	30	20	19	–	64
		*Bb*	25	–	2	4	22	29	21	3	51
Filamin-A (isoform 1 & 2)	NP_001447.2 NP_001104026.1	None	–	22	11	–	27	–	3	12	–
		LTA	–	16	16	11	24	–	4	–	–
		*Bb*	2	26	15	10	24	–	6	1	–
Gelsolin (isoform a-g)	NP_000168.1	None	–	–	2	–	28	13	–	1	34
		LTA	–	–	2	–	28	32	–	–	28
		*Bb*	1	–	1	–	28	25	–	–	32
Hemoglobin subunit α	NP_000508.1	None	–	–	8	–	24	10	–	–	24
		LTA	–	–	11	–	16	25	9	–	17
		*Bb*	–	–	10	–	20	22	20	–	32
HLA class I A-1 α chain A*03:01:0:01 precursor	NP_002107.3	None	–	7*	18*	–	44*	7*	–	2*	16*
		LTA	–	12*	25*	–	42*	27*	–	–	20*
		*Bb*	6*	13*	21*	12*	48*	19*	3	–	12*
HLA class I Cw-1 α chain precursor	NP_002108.4	None	–	10*	25*	-	55*	8*	–	4	21*
		LTA	–	–	38*	14*	55*	20*	–	–	25*
		*Bb*	6*	14*	33*	10*	58*	14*	3	–	14*
HLA class II DR α chain precursor	NP_061984.2	None	10	24	18	–	31	7*	4	21	32
		LTA	–	20	28	21	34	17	18	14	33
		*Bb*	11	22	13	13	20	10	11	19	20
Integrin α-M (isoform 1 & 2)	NP_000623.2 NP_001139280.1	None	27*	12	18	–	50	24*	1	18	77*
		LTA	–	7	37	12*	48	54*	8	2	94
		*Bb*	71*	14	23	17*	72	93*	5	10	84
Macrophage mannose receptor 1 precursor	NP_002429.1	None	–	–	4	–	23	1	3	–	33
		LTA	–	–	7	–	15	5	12	–	36
		*Bb*	1	1	4	8	28	20	22	–	40
MHC class I B precursor	NP_005505.2	None	1	10*	26*	–	–	11*	–	4	25*
		LTA	–	13*	41*	11*	–	32*	8	–	28*
		*Bb*	7*	14*	32*	7*	–	25*	3	–	21*
Transferrin receptor protein (isoform 1–3)	NP_003225.2	None	4	–	–	–	9	–	–	1	11
		LTA	–	–	–	6	15	3	–	–	19
		*Bb*	16	6	4	33	25	34	2	–	47
TGF-ß-induced protein ig-H3	NP_000349.1	None	6	10	2	–	26	4	2	15	33
		LTA	–	7	4	9	21	10	1	–	21
		*Bb*	8	10	–	10	14	9	2	–	13
Vimentin	NP_003371.2	None	4	7	34	–	105	12	4	24	41
		LTA	–	7	39	6	105	6	11	3	40
		*Bb*	16	3	35	2	125	6	12	9	33

* *Denotes that self-peptides could be derived from multiple related isoforms*.

### Overlapping Functional Annotation Clustering of Parent Proteins

**Figure 5:**
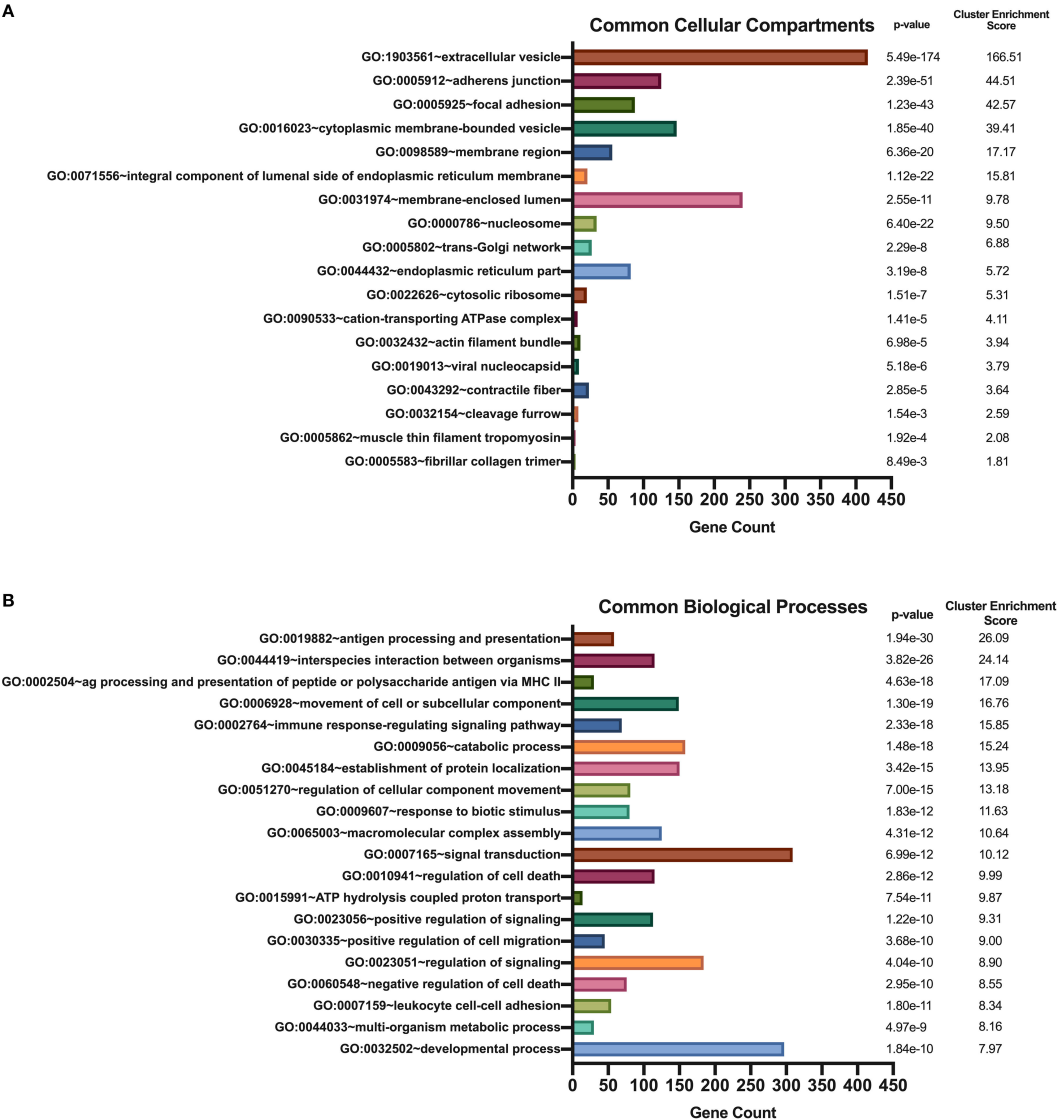
Enriched Gene Ontology terms derived from source proteins identified in all stimuli. DAVID functional annotation clustering of cellular compartments **(A)** and biological processes **(B)** identified in all healthy donors' mo-DCs that were left at rest or stimulated with LTA or live *B. burgdorferi* for 24 h. RefSeq Protein Accession numbers were converted to UniProt gene identifiers for DAVID compatibility. GraphPad Prism version 8.4.2 was used for graphical representation.

We used DAVID's functional annotation tool to cluster parent proteins identified from all donors and all stimulation conditions into biologically similar terms (16, 17). Accordingly, unique RefSeq protein accession numbers for identified source proteins were converted into UniProt accession identifiers that were categorized into shared cellular compartments and biological processes (15). First, we analyzed those 2,460 proteins that were common peptide sources in all conditions (Figure 4). Conversion of these 2,460 protein identifiers to gene annotations added redundancy to the listing as a result of protein isoforms. Consequently, we removed all duplicate genes and analyzed 1,852 unique gene identifiers. Binning of gene sets into cellular compartments resulted in 18 functional annotation clusters (Figure 5A). Top clusters were enriched with genes found in extracellular vesicles or exosomes; cell-cell adherens or anchoring junctions; focal adhesions; cytoplasmic, membrane-bound vesicles; membrane regions, microdomains or rafts; and integral and intrinsic components of the lumenal side of the endoplasmic reticulum membrane. Similar settings were used for clustering of gene sets into biological processes, yielding 186 functional annotation clusters (Figure 5B). Top clusters in this enrichment analysis included antigen processing and presentation; interspecies interaction between organisms or multi-organism cellular processes; antigen processing and presentation of peptide or polysaccharide antigen via MHC class II, movement of cell or subcellular components; immune response-regulating signaling pathways; catabolic processes; and establishment of protein localization or protein transport. Notably, enriched cellular compartments reflect the biological processes commonly taking place inside the cell. For example, antigen processing and presentation of peptides, which takes place in cytoplasmic, membrane-bound vesicles and with molecules that are integral components of the lumenal side of the endoplasmic reticulum membrane, was the top biological process among all donors and all stimuli. These results strongly suggest that common biological processes in mo-DCs require extensive cellular membrane involvement from numerous compartments.

### Common and Unique Functional Annotation Clustering of Parent Proteins Under Different Conditions

**Figure 6:**
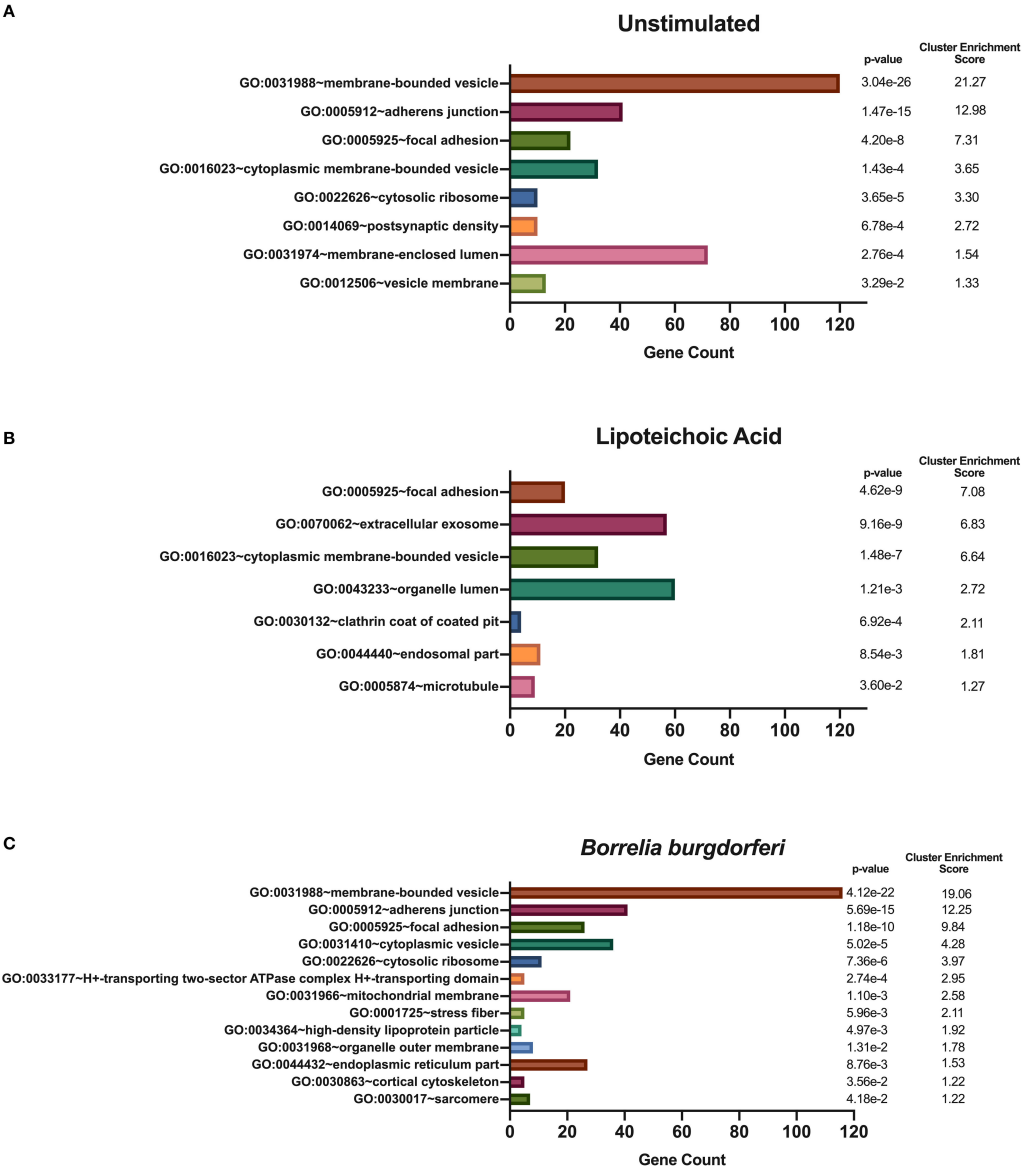
Enriched cellular compartments derived from source proteins identified exclusively in each individual stimulus. DAVID functional annotation clustering of cellular compartments identified in mo-DCs that were left at rest **(A)** or stimulated with LTA **(B)** or live *B. burgdorferi* **(C)** for 24 h. RefSeq Protein Accession numbers were converted to UniProt gene identifiers for DAVID compatibility. GraphPad Prism version 8.4.2 was used for graphical representation.

We hypothesized that exposure of mo-DCs to *B. burgdorferi* will alter processing and presentation by HLA-DR molecules in dendritic cells, eliciting changes in the immunopeptidome by triggering distinct biological processes and involving cellular compartments exclusive to the cellular response to *B. burgdorferi*. We used DAVID's functional annotation tool to identify cellular compartments and biological processes exclusive to unstimulated cells, LTA-stimulated mo-DCs or live *B. burgdorferi*-stimulated mo-DCs (Figure 6). Conversion of RefSeq Protein Accession numbers to UniProt gene annotations reduced the number of identified source proteins from 1,048 to 672 genes in unstimulated mo-DCs, from 730 proteins to 435 genes in LTA-stimulated cells, and from 1,020 proteins to 653 genes in *B. burgdorferi*-stimulated mo-DCs. The most significant functional annotation clusters enriched in LTA- and live *B. burgdorferi*-stimulated mo-DCs overlap with those found in all donors regardless of stimuli. These clusters include membrane-bound or extracellular vesicles or exosomes; adherens or anchoring junctions; focal adhesions; and cytoplasmic, membrane-bounded vesicles (Figure 6). Interestingly, the genes enriched in these clusters differ from those identified in unstimulated mo-DCs. Furthermore, in LTA-stimulated mo-DCs involvement of clathrin coat of coated pits or clathrin-coated endocytic vesicles; the endosomal or vacuolar membrane; and microtubules were uniquely enriched (Figure 6B). On the other hand, *B. burgdorferi* stimulation elicited involvement of other cellular compartments such as the proton-transporting two-sector ATPase complex; the mitochondrial membrane; high-density lipoprotein or plasma lipoprotein particles; organelle or mitochondrial outer membrane; and the cell cortical cytoskeleton (Figure 6C). These results suggest that *B. burgdorferi* stimulation of dendritic cells leads to utilization and subsequent sampling of unique compartments within the cell that become major sources of self-peptides.

**Figure 7:**
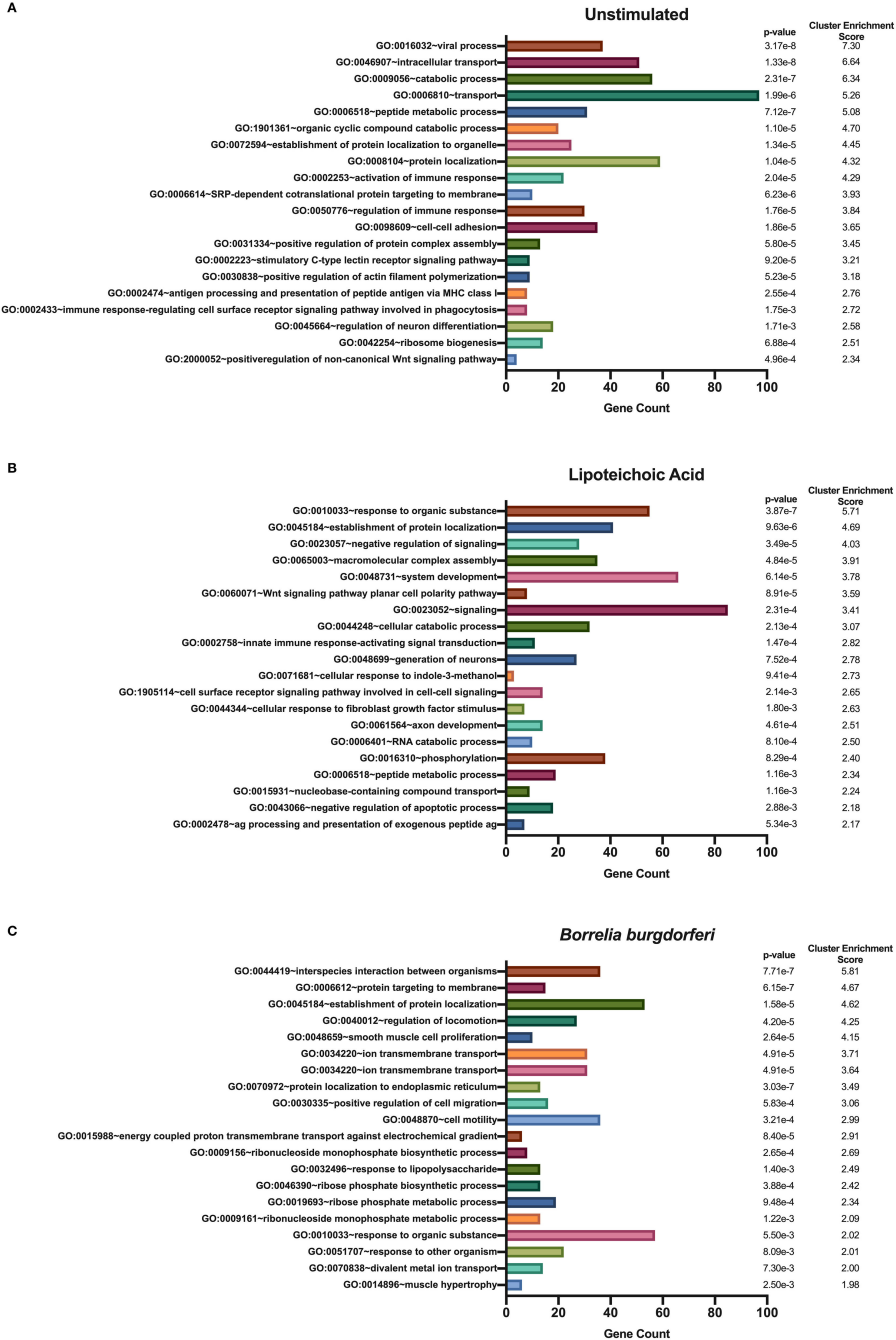
Enriched biological processes derived from source proteins identified exclusively in each individual stimulus. DAVID functional annotation clustering of biological processes identified in mo-DCs that were left at rest **(A)** or stimulated with LTA **(B)** or live *B. burgdorferi* **(C)** for 24 h. RefSeq Protein Accession numbers were converted to UniProt gene identifiers for DAVID compatibility. GraphPad Prism version 8.4.2 was used for graphical representation.

We used DAVID's functional annotation tool to identify unique biological processes in LTA- and in *B. burgdorferi*-stimulated mo-DCs (Figure 7). Consistent with previous results, several gene ontology terms in the top annotation clusters identified in both conditions overlap with those enriched in the all donors and all conditions set. However, the genes comprising these clusters differ from those observed in the all donors and all stimuli grouping. Biological processes unique to LTA-stimulated mo-DCs include intracellular protein transport; positive regulation of the immune response; phosphate-containing compound metabolic processes; cellular response to indole-3-methanol; and nucleobase-containing compound transport. Interestingly, enriched clusters unique to *B. burgdorferi*-stimulated mo-DCs include ion transmembrane transport; ribosome biogenesis; ncRNA processing; ncRNA metabolic processes; response to lipopolysaccharide; organophosphate biosynthetic processes; ribonucleotide metabolic processes, and muscle hypertrophy and adaptation. These results lend additional support that *B. burgdorferi* stimulation of DCs leads to utilization and subsequent sampling of unique cellular compartments and pathways for processing and presentation on MHC class II molecules.

### Autoantigens in Lyme Arthritis

Previous studies identified peptides from annexin A2, apolipoprotein B-100, thymidine phosphorylase (endothelial cell growth factor), and stromelysin-2 (matrix metalloproteinase 10) as targets of autoreactive T cells in a subset of human Lyme disease patients (22–24). We interrogated our data to determine whether these proteins were sources of HLA-DR-binding peptides and identified peptides from annexin A2, apolipoprotein B-100 and thymidine phosphorylase. Our analysis showed that all donors presented peptides from annexin A2, with five out of nine donors presenting peptides regardless of stimulus or HLA-DR genotype (Supplementary Figure 2). We identified 107 unique (498 total) annexin A2 peptides, including nested peptide sets containing the predicted promiscuous HLA-DR-binding sequences ^50^GVDEVTIVNILTNRSNAQR^68^ and ^164^SGDFRKLMVALAKGRRA^180^, along with peptide clusters from other regions within the protein (Supplementary Figure 2). Our analysis did not identify a previously reported sequence (^285^DKVLIRIMVSRSEVD^299^) found to be T cell reactive in patients with Lyme arthritis (24).

**Figure 8:**
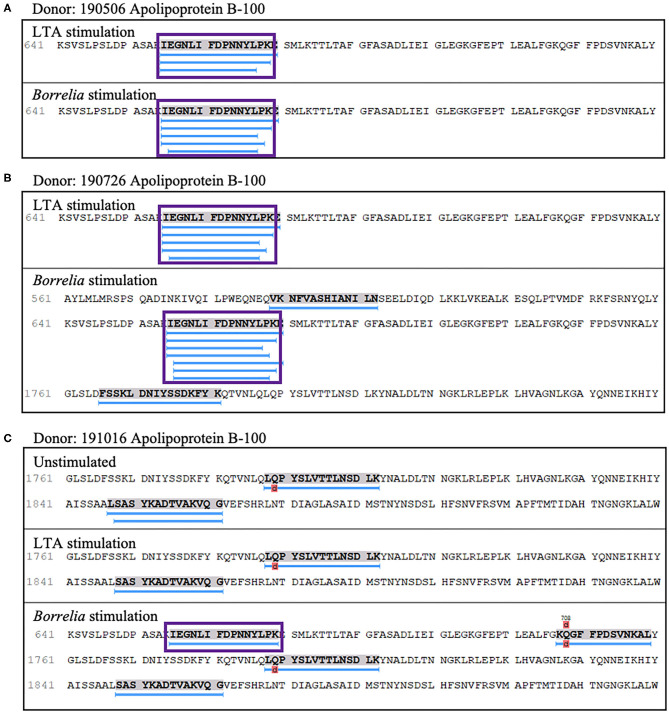
Apolipoprotein B-100 presentation is associated with LTA and *Borrelia* stimulation. **(A)** Representative region of apolipoprotein B-100 previously reported as an autoantigenic epitope (purple rectangle) detected in mo-DCs from healthy donor 190506 expressing HLA-DRB1*03:01/03:02 stimulated with LTA (top panel) or *B. burgdorferi* (bottom panel) and healthy donor 190726 expressing HLA-DRB1*03:01/07:01 **(B)**. **(C)** Representative autoantigenic epitope of apolipoprotein B-100 detected in mo-DCs from healthy donor 191016 expressing HLA-DRB1*01:01/04:01 stimulated with *B. burgdorferi* (bottom panel).

We also detected peptides from apolipoprotein B-100 in seven out of nine donors. Interestingly five donors (donors 190506, 190726, 191114, 191202, and 200218), expressing *HLA-DRB1* alleles 03:01/03:02, 03:01/07:01, 01:01/16:01, 11:01/15:01, and 01:01/15:01 respectively, presented peptides from this protein only when stimulated with LTA or live *B. burgdorferi*. We found 22 unique (58 total) apolipoprotein B-100 peptides. Included were nested sets of the peptide ^655^IEGNLIFDPNNYLPK^669^, which was previously identified in the synovial tissue in a patient with antibiotic-refractory Lyme arthritis (HLA-DRB1^*^03:01/03:05 alleles) (23), and peptides from other regions within the protein (Figures 8A–C).

**Figure 9:**
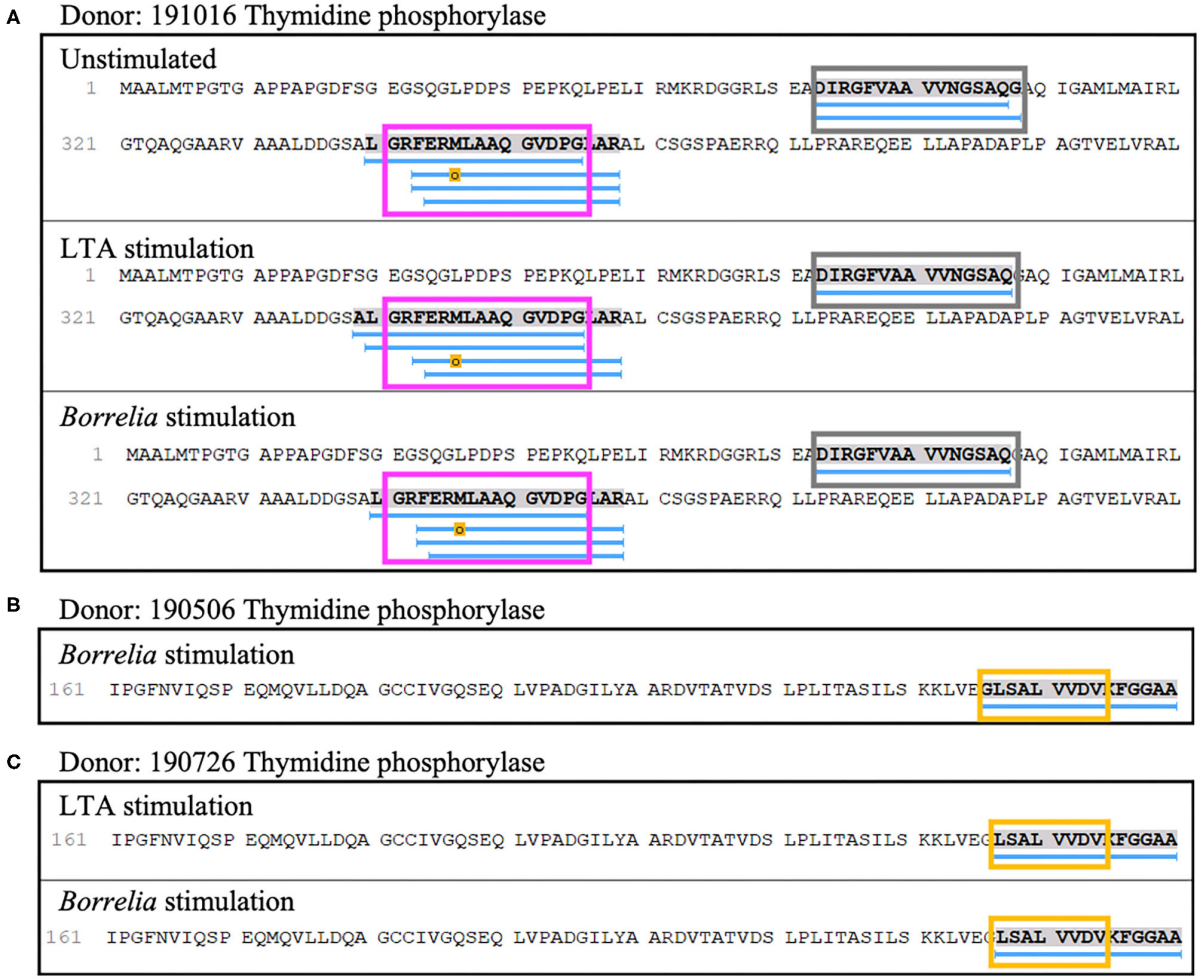
Thymidine phosphorylase presentation is associated with LTA and *Borrelia* stimulation. **(A)** Representative region of thymidine phosphorylase previously reported as an autoantigenic epitope (pink rectangle) identified in mo-DCs at rest (top panel), stimulated with LTA (middle panel) or *B. burgdorferi* (bottom panel) from healthy donor 191016 expressing HLA-DRB1*01:01/04:01. A previously predicted promiscuous binding sequence (gray rectangle) was also detected in this donor. **(B)** Partial representative region of thymidine phosphorylase previously reported as an autoantigenic epitope (yellow rectangle) identified in mo-DCs stimulated with *B. burgdorferi* from healthy donor 190506 expressing HLA-DRB1*03:01/03:02. **(C)** Partial autoantigenic epitope was also detected in healthy donor 190726 expressing HLA-DRB1*03:01/07:01.

Lastly, four out of nine donors (donors 190506, 190726, 191016, and 200218), expressing *HLA-DRB1* alleles 03:01/03:02, 03;01/07:01, 01:01/04:01, and 01:01/15:01 respectively, presented 8 unique (23 total) peptides from thymidine phosphorylase. The nested sets of peptides included the previously predicted HLA-DR-binding sequence A^52^DIRGFVAAVVNSAQGAQI^71^ and the peptide L^340^GRFERMLAAQGVDPG^355^ (Figure 9A), which was previously identified in a patient with antibiotic-refractory Lyme arthritis (HLA-DRB1^*^01:01 alleles) (22). Notably, we did not find the predicted S^220^KKLVEGLSALVVDV^234^ peptide, but did identify a smaller overlapping sequence (Figures 9B,C).

## Discussion

The observation that *B. burgdorferi*-induced changes led to robust expression of HLA-DR on the surface of monocyte-derived dendritic cells in a time- and dose-dependent manner led us to reason that the HLA-DR associated self-immunopeptidome was altered, and formed the premise for this study. Using the natural processing and presentation capabilities of mo-DCs, we isolated sufficient peptide-HLA-DR complexes (13) to define the self-immunopeptidome at steady state, and its variation during stimulation with the TLR-2 ligand lipoteichoic acid or with live *B. burgdorferi*. In all conditions, we isolated peptides that varied in length (seven to 65 amino acids long) (25) averaging 15 residues, the optimal length of MHC class II presented antigens. Commonly presented peptides were found in nested sets, with their predicted core matching binding motifs associated with the donors' *HLA-DRB1* expressed alleles (20). Isolated peptides were mostly derived from proteins associated with membrane bound compartments, including the MHC II compartment and its cargo, the cytoskeleton, and proteins involved in cell adhesion and migration (Figure 3). Overall, the isolated peptides exhibited bona fide characteristics attributed to MHC Class II processing. Therefore, we believe that the class II processing and presentation pathway was fully functional in mo-DCs under the conditions studied.

The top biological process in mo-DCs irrespective of stimulus was antigen processing and presentation, clearly highlighting the importance of this process in the function of mo-DCs. Genes encoding proteins enriched in this GO term include pathogen recognition molecules such as *cd1b* (P29016), *cd1c* (P29017), and *cd209* (Q9NNX6), cathepsins (*ctsd* (P07229), *ctsh* (P09668), *ctsl* (P07711), and *ctss* (P25774), *cd74* (P04233), major histocompatibility complex, class II (*hla-dm, hla-do, hla-dp, hla-dq, hla-dr*) and class I molecules (*hla-a* (P04439), *hla-b* (P01889), *hla-c* (P10321), *hla-e* (P13747), *hla-f* (P30511), *hla-g* (P17693), and proteasome subunits (*psmd1* (Q99460), *psma3* (Q43242), *psma7* (P51665), *psmb1*(P20618) among others (Supplementary List 1). Interspecies interaction between organisms and antigen processing and presentation of peptide or polysaccharide antigen *via* MHC class II were also top clusters in shared biological processes between mo-DCs regardless of stimulus, again emphasizing the significance of these processes during both steady state and pathogenic challenge.

Interestingly, the immunopeptidome was significantly altered following stimulation with whole viable *B. burgdorferi*. This was evident not only in the identified peptides, but also in the source proteins that clustered to specific biological processes. Accordingly, the most significant biological process in *B. burgdorferi*-stimulated mo-DCs involved proteins that participate in interspecies interaction between organisms. These proteins are absent from the cluster in the shared biological processes, thus are unique to *Borrelia*-stimulated mo-DCs, and have been implicated in the immune response against pathogens, including *ccr5* (26) (P51681), *stat3* (P40763), *tnfaip3* (27) (P21580), *icam1* (P05362), and *cd86* (P42081) among others (Supplementary List 2). Identification of these peptides as part of the HLA-DR immunopeptidome from *Borrelia*-stimulated mo-DCs provides insights into novel biological process relevant to human Lyme disease. For example, potential involvement of the C-C motif chemokine receptor 5 (CCR5) in the interaction of *B. burgdorferi* and mo-DCs provides a novel pathway for early cytokine production during challenge with the *Borrelia* spirochete. Notably, engagement of CCR5 by the HIV-1 glycoprotein gp120 leads to increased production of interleukin-6 (IL-6), resulting in dysregulation of STAT3 signaling and subsequent impairment of DC functionality (28). In addition, aberrant TNFAIP3 signaling also leads to increased IL-6 production in dendritic cells, implicating TNFAIP3 in the development of autoimmunity in dendritic cells as well as B-cells (27). IL-6 expression has been shown to be upregulated in patients with acute Lyme disease and remain elevated months after antibiotic treatment (29), suggesting that increased IL-6 levels in Lyme patients may play a role in the development of chronic symptoms. Thus, future studies aimed at elucidating the roles of IL-6, CCR5, STAT3, and TNFAIP3, potential cross-talk between these signaling pathways and how these may affect functionality of dendritic cells during *B. burgdorferi* challenge should be explored.

The top biological process in LTA-stimulated mo-DCs was response to organic substances. Genes associated with this GO term include *cd63* (P08962) and *mapkapk2* (P49137). CD63 is a cell surface-associated receptor that can be found in endosomes internalized into the cell *via* clathrin-dependent endocytosis (30), the known route of internalization of LTA (31). MAPKAPK2, a pro-inflammatory effector kinase, has been implicated in the innate immune response (32), signaling downstream of p38α, thus offering a differential response than that undertaken after *B. burgdorferi* stimulation. Further, viral process was the most significant biological process in mo-DCs at steady state, with none of the above-mentioned genes represented in this GO term. Other genes such as *cd46* (complement inhibitor; P15529) (33), *il10rb* (Q08334) (34), *ifnar2* (P48551), and *tgfb1*(P01137) that code for proteins implicated in viral immunity were enriched in this GO term, suggesting that antiviral processes are a main focus for mo-DCs at rest. Collectively, these findings reveal that the HLA-DR-bound self-immunopeptidome presented by mo-DCs is dynamic in nature and changes with the activation state of the cell reflecting differential functional capabilities. These studies will form the basis for future work investigating how *B. burgdorferi* impacts the function of mo-DCs, a cell type known to drive early innate and adaptive immune processes that inarguably impact the ability of the host to control infection.

Genetic susceptibility to autoimmune diseases is strongly associated with specific HLA alleles (35). A significant number of individuals with untreated Lyme disease will develop Lyme arthritis, a condition largely responsive to antibiotic treatment, but a subset of patients suffer from long term inflammation or antibiotic-refractory Lyme arthritis. In antibiotic-refractory Lyme arthritis, a late manifestation of *B. burgdorferi* infection and a condition with autoimmune components, the *HLA-DRB1* alleles 01:01, 04:01, and 15:01 are genetically linked to disease pathogenesis (36). Yet, presentation of autoantigenic peptides in Lyme arthritis by donors in our healthy cohort with risk HLA alleles, suggests that it is unlikely these alleles alone are responsible for the onset of disease. Rather, a breakdown in tolerance to self-antigens, triggered by infection with *B. burgdorferi*, may contribute to the development of dysregulated immunity and ultimately Lyme arthritis. Relevant to this study is that several proteins have been identified as targets of self-reactive CD4^+^ T cells and autoantibodies in Lyme arthritis. These proteins were also identified using a mass spectrometry based approach, identifying HLA-DR associated peptides uniquely expressed in inflamed synovial tissue from patients with Lyme arthritis (22–24). Interestingly, we isolated peptides derived from three of these proteins: annexin A2, apolipoprotein B-100 and thymidine phosphorylase (endothelial cell growth factor).

Annexin A2, a Ca^2+^-regulated membrane binding protein with key roles in membrane-cytoskeleton and membrane-membrane binding events, has been shown to be an autoantigen in several immune-mediated diseases including anti-phospholipid syndrome and rheumatoid arthritis (RA) (24). We found that annexin A2 was a major peptide donor in all subjects under all conditions. Among the peptides we isolated were two sequences identified by others as T cell targets and shown to be promiscuous HLA-DR binders (24). This feature is consistent with our finding that these peptides were isolated in all our subjects regardless of *HLA-DR* allele. A previous model was presented to explain how annexin A2 may become immunogenic and contribute to autoimmunity (24). In this model the “first hit” was driven by the presence of the spirochete, which facilitated annexin A2 uptake, processing, and presentation. Our results identifying auto-immunogenic annexin A2 peptides bound to HLA-DR in unstimulated mo-DCs implies that this first hit is spirochete independent. We hypothesize that mo-DCs, which appear to be constitutively processing and presenting annexin A2 peptides, are driving tolerance in a low-level self-reactive T cell population that is well-regulated, likely in the secondary lymphoid tissue. These cells can then be activated, as previously proposed (2nd hit), when target tissues upregulate autoantigen expression in an inflammatory setting, allowing local APCs to drive this self-reactive T cell population to expand and differentiate into effector cells. As noted previously, annexin A2 is upregulated in the inflamed joints of Lyme arthritis patients (24).

Peptides derived from thymidine phosphorylase (endothelial cell growth factor) were also identified in four of nine subjects in our study. Among the peptides isolated are two previously identified autoreactive, promiscuous binders in subjects expressing *HLA-DRB1* alleles 03:01/03:02 and 03:01/07:01, which are not considered risk alleles in Lyme arthritis (22). Notably, we did not identify the two non-promiscuous HLA-DRB1^*^01:01 and 04:01 binders in subjects expressing those alleles, irrespective of the presence of live *Borrelia*. This finding suggests that these peptides may become immunogenic only in inflamed tissue. It is unknown whether thymidine phosphorylase is expressed by mo-DCs, yet its expression is upregulated in many malignancies, as well as during tissue regeneration and repair where it functions as a potent angiogenesis factor (37). Also, expression of thymidine phosphorylase is uniquely present in the synovial fluid and synovial tissue of patients with Lyme arthritis (22). This suggests that other factors including, but not limited to localized tissue expression levels and the presence of potent APCs, as well as recruitment of T cells into inflamed tissue *via* chemokines, all of which are known to occur in the joint of subjects with Lyme arthritis, may drive neo-antigenicity of thymidine phosphorylase peptides ultimately leading to the activation of self-reactive T cells.

Apolipoprotein B-100 (apoB-100) is a major protein source of autoantigenic peptides in rheumatoid arthritis, atherosclerosis, systemic lupus erythematosus (SLE), and Lyme arthritis (23). Specifically, peptides p45 and p210 corresponding to residues 661–680 and 3,136–3,155, respectively in the protein, have been implicated in the immunogenicity of apoB-100 (38). Healthy donors in our cohort presented nested sets of the autoantigenic Lyme arthritis peptide ^655^IEGNLIFDPNNYLPK^669^, which overlaps with the p45 peptide from residue 661–669 (FDPNNYLPK). Accordingly, we speculate that this sequence overlap identifies the minimal autoreactive peptide. Speculation surrounding potential mechanisms responsible for local loss of tolerance to apoB-100 suggest that Th1 responses at sites of inflammation supply the necessary signals for increased expression of co-stimulatory molecules, leading to increased antigen presentation and ultimate loss of tolerance (39). Given the central role dendritic cells play in driving T cell responses in secondary lymphoid compartments, it is tempting to speculate that specific self-peptides become immunogenic, under infection-induced or inflammatory conditions, driving dysregulated adaptive immune responses that breach the threshold of established tolerance mechanisms.

One of the most commonly presented proteins in our assay was vimentin, a member of the intermediate filament family of the cellular cytoskeleton, which plays a crucial role in the pathogenesis of various inflammatory and autoimmune diseases including: rheumatoid arthritis, systemic lupus erythematosus, ankylosing spondyloarthritis, among others (40). Antigenic hallmarks of vimentin, among other recognized autoantigens in autoimmune diseases, are exacerbated by post translational modifications, specifically citrullination. The role of protein citrullination in the context of Lyme arthritis remains relatively unexplored and deserves future examination, given that the *HLA-DRB1* alleles 01:01 and 04:01, risk alleles in Lyme arthritis, contain the shared epitope, a five-residue sequence in the HLA-DRβ chain associated with severe rheumatoid arthritis (41). In addition, the function and abundance of vimentin and other autoantigens have been implicated in their potential to become immunogenic in inflammatory settings, warranting further exploration.

Overall, our study contributes novel insights to understanding the interaction between dendritic cells and the *B. burgdorferi* spirochete. Our results corroborated known aspects of class II MHC presentation, profiled the HLA-DR self-immunopeptidome presented during *Borrelia* challenge, and identified sets of unique self-peptides derived from proteins associated with distinct biological processes and cellular compartments following exposure of mo-DCs to the live spirochete. Importantly, we advanced our understanding of the biological processes occurring in dendritic cells from healthy donors during *Borrelia* infection, which may shed light into mechanisms that promote the range of disease outcomes, including Lyme arthritis and PTLDS.

## Data Availability Statement

The list of all self-peptides identified under all conditions is provided as Supplemental Material (Supplementary List 3). Peaks files and .raw files are stored on a secure Johns Hopkins University One Drive server and will be made available to interested investigators. Please contact the corresponding author to obtain access.

## Ethics Statement

Ethical review and approval was not required for the study on human participants in accordance with the local legislation and institutional requirements. Written informed consent for participation was not required for this study in accordance with the national legislation and the institutional requirements.

## Author Contributions

MG-H and MS contributed to the conception and design of the study. ET and ED contributed to the conception of the natural antigen processing assay. RC and RO'M contributed to optimization and collection of LC-MS/MS data. All authors contributed to manuscript preparation as well as read and approved the submitted version.

## Conflict of Interest

The authors declare that the research was conducted in the absence of any commercial or financial relationships that could be construed as a potential conflict of interest.
