# Supplementary material for: Borrelia burgdorferi-Induced Changes in the Class II Self-Immunopeptidome Displayed on HLA-DR Molecules Expressed by Dendritic Cells
Source: Front Med (Lausanne). 2020 Sep 16;7:568. doi: 10.3389/fmed.2020.00568 (PMC7524959; doi:10.3389/fmed.2020.00568)
Supplement: Supplementary file 1 [file Presentation_1.pdf]

Supplementary Material

1 Supplementary Data

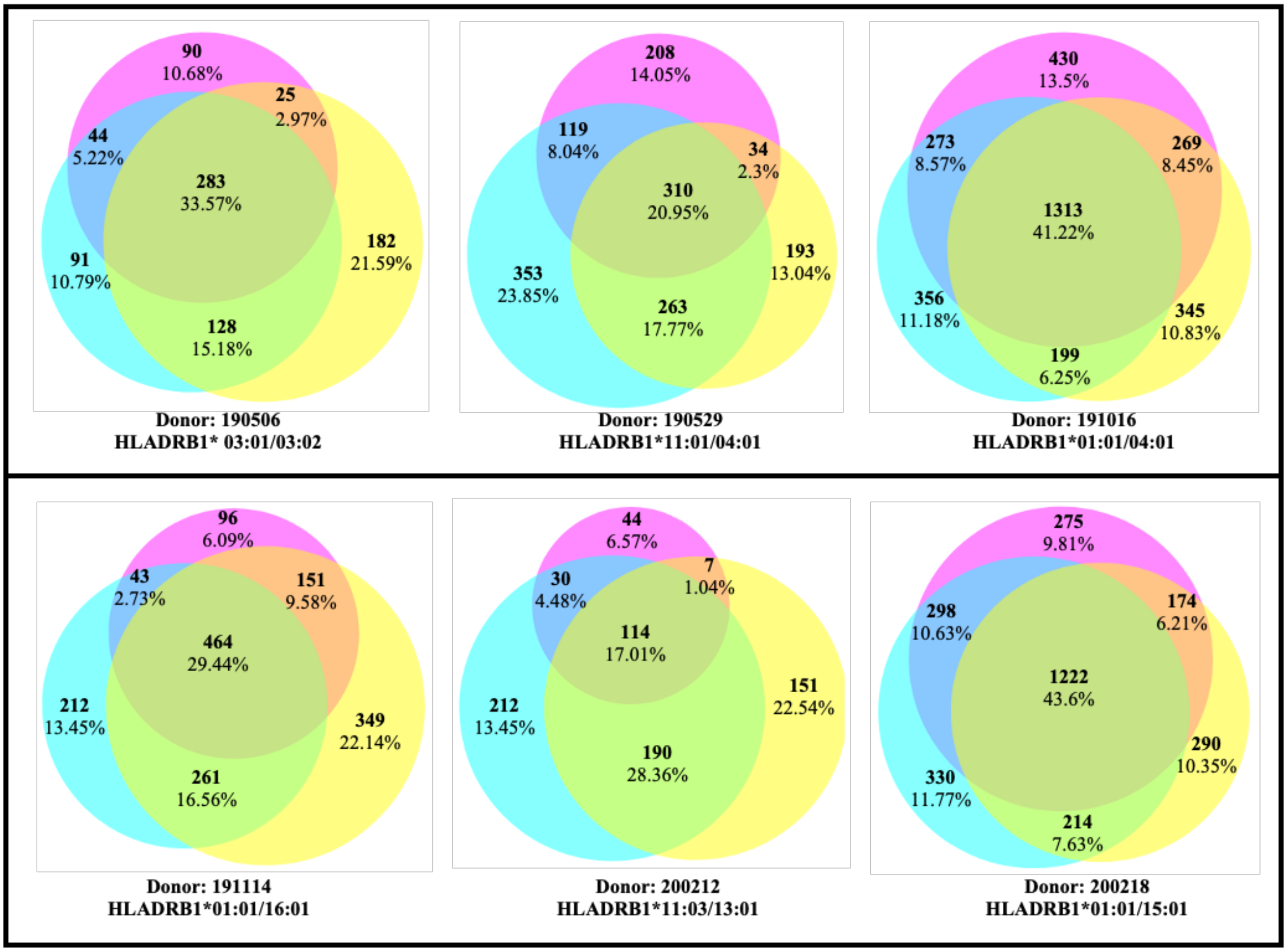

Supplementary Figure 1. Common and exclusive parent proteins presented per donor. Representative BioVenn diagrams illustrating overlap and exclusivity in source proteins identified by LC-MS/MS from mo-DCs left at rest (fuchsia circle) or stimulated with LTA (aqua circle) or live *B. burgdorferi* (yellow circle) for 24 hours.

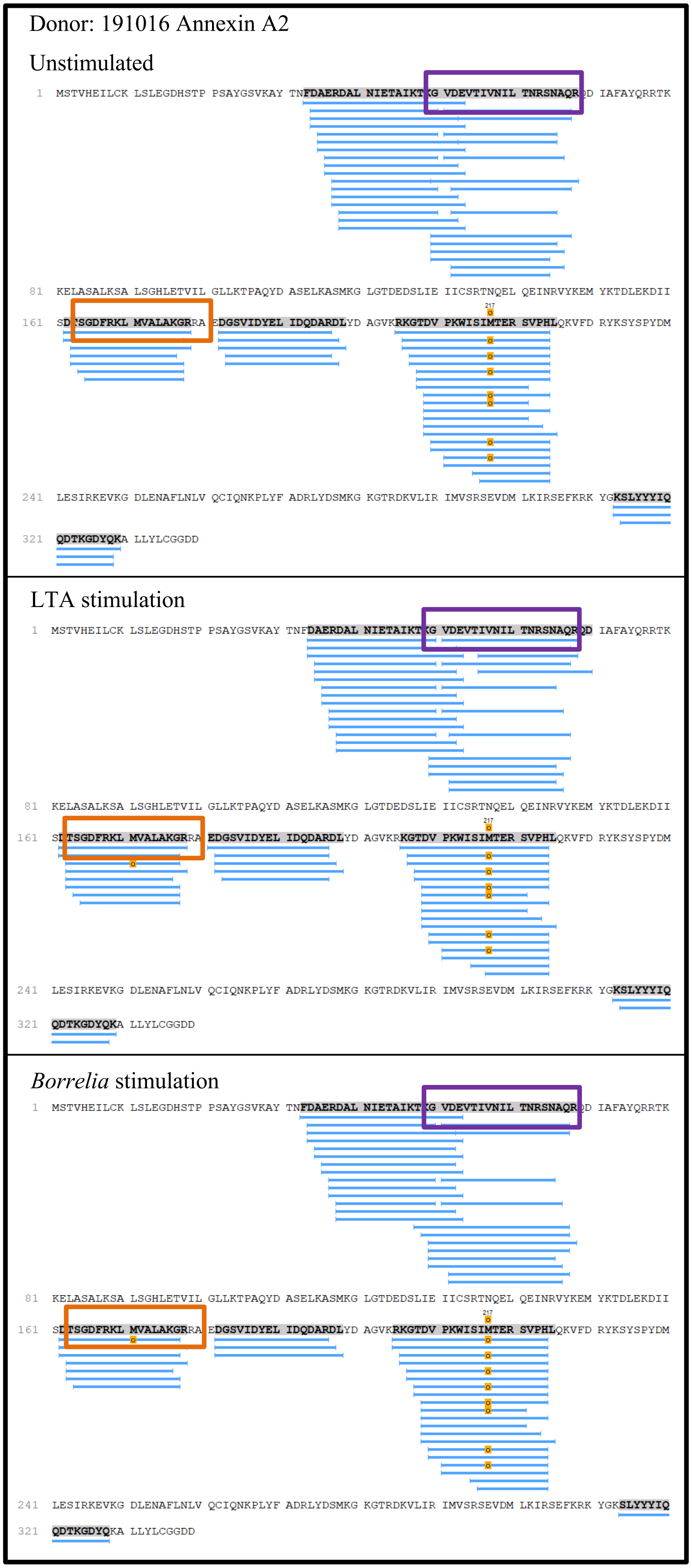
Donor: 191016 Annexin A2
Unstimulated
1 MSTVHEILCK LSLEGDHSTP PSAYGSVKAY TNFDAERDAL NIETAIKTKG VDEVTIVNIL TNRSNAQRQD IAFAYQRRTK
81 KELASALKSA LSGHLETVIL GLLKTPAQYD ASELKASMKG LGTDEDSLIE IICSRTNQEL QEINRVYKEM YKTDLEKDII
217
161 SDTSGDFRKL MVALAKGRRA EDGSVIDYEL IDQDARDLYD AGVKRKGTDV PKWISIMTER SVPHLQKVFD RYKSYSPYDM
241 LESIRKEVKG DLENAFLNLV QCIQNKPLYF ADRLYDSMKG KGTRDKVLIR IMVSRSEVDM LKIRSEFKRK YGKSLYYYIQ
321 QDTKGDYQKA LLYLCGGDD
LTA stimulation
1 MSTVHEILCK LSLEGDHSTP PSAYGSVKAY TNFDAERDAL NIETAIKTKG VDEVTIVNIL TNRSNAQRQD IAFAYQRRTK
81 KELASALKSA LSGHLETVIL GLLKTPAQYD ASELKASMKG LGTDEDSLIE IICSRTNQEL QEINRVYKEM YKTDLEKDII
217
161 SDTSGDFRKL MVALAKGRRA EDGSVIDYEL IDQDARDLYD AGVKRKGTDV PKWISIMTER SVPHLQKVFD RYKSYSPYDM
241 LESIRKEVKG DLENAFLNLV QCIQNKPLYF ADRLYDSMKG KGTRDKVLIR IMVSRSEVDM LKIRSEFKRK YGKSLYYYIQ
321 QDTKGDYQKA LLYLCGGDD
Borrelia stimulation
1 MSTVHEILCK LSLEGDHSTP PSAYGSVKAY TNFDAERDAL NIETAIKTKG VDEVTIVNIL TNRSNAQRQD IAFAYQRRTK
81 KELASALKSA LSGHLETVIL GLLKTPAQYD ASELKASMKG LGTDEDSLIE IICSRTNQEL QEINRVYKEM YKTDLEKDII
217
161 SDTSGDFRKL MVALAKGRRA EDGSVIDYEL IDQDARDLYD AGVKRKGTDV PKWISIMTER SVPHLQKVFD RYKSYSPYDM
241 LESIRKEVKG DLENAFLNLV QCIQNKPLYF ADRLYDSMKG KGTRDKVLIR IMVSRSEVDM LKIRSEFKRK YGKSLYYYIQ
321 QDTKGDYQKA LLYLCGGDD

Supplementary Figure 2. Annexin A2 is presented in all donors and all stimuli. Representative regions of annexin A2 previously predicted to be promiscuous HLA-DR binding sequences (purple and orange rectangles) identified in mo-DCs at rest (top panel), stimulated with LTA (middle panel) or *B. burgdorferi* (bottom panel) from healthy donor 191016 expressing HLA-DRB1*01:01/04:01.
